# Inhibition of Glucose-6-Phosphate Dehydrogenase Could Enhance 1,4-Benzoquinone-Induced Oxidative Damage in K562 Cells

**DOI:** 10.1155/2016/3912515

**Published:** 2016-08-31

**Authors:** Juan Zhang, Meng Cao, Wenwen Yang, Fengmei Sun, Cheng Xu, Lihong Yin, Yuepu Pu

**Affiliations:** Key Laboratory of Environmental Medicine Engineering of Ministry of Education, School of Public Health, Southeast University, Nanjing 210009, China

## Abstract

Benzene is a chemical contaminant widespread in industrial and living environments. The oxidative metabolites of benzene induce toxicity involving oxidative damage. Protecting cells and cell membranes from oxidative damage, glucose-6-phosphate dehydrogenase (G6PD) maintains the reduced state of glutathione (GSH). This study aims to investigate whether the downregulation of G6PD in K562 cell line can influence the oxidative toxicity induced by 1,4-benzoquinone (BQ). G6PD was inhibited in K562 cell line transfected with the specific siRNA of G6PD gene. An empty vector was transfected in the control group. Results revealed that G6PD was significantly upregulated in the control cells and in the cells with inhibited G6PD after they were exposed to BQ. The NADPH/NADP and GSH/GSSG ratio were significantly lower in the cells with inhibited G6PD than in the control cells at the same BQ concentration. The relative reactive oxygen species (ROS) level and DNA oxidative damage were significantly increased in the cell line with inhibited G6PD. The apoptotic rate and G2 phase arrest were also significantly higher in the cells with inhibited G6PD and exposed to BQ than in the control cells. Our results suggested that G6PD inhibition could reduce GSH activity and alleviate oxidative damage. G6PD deficiency is also a possible susceptible risk factor of benzene exposure.

## 1. Introduction

Glucose-6-phosphate dehydrogenase (G6PD) deficiency, an X-linked genetic disorder, is a common human enzymopathy affecting over 400 million individuals worldwide [1]. Individuals with G6PD deficiency can experience neonatal jaundice and acute hemolysis when they are exposed to oxidative stress induced by various factors, such as drugs, infections, or foods (e.g., fava beans), although affected individuals are asymptomatic [2].

G6PD, which is the rate-limiting enzyme of the pentose phosphate pathway, converts glucose-6-phosphate into 6-phosphogluconolactone and maintains the level of nicotinamide dinucleotide hydrogen phosphate (NADPH), which in turn promotes glutathione (GSH) regeneration; as a consequence, cells are protected against oxidative damage and injury [3]. Under G6PD-deficient conditions and oxidative stress, residual G6PD is possibly inadequate to inhibit large amounts of ROS and to prevent severe hemolysis [4].

Oxidative damage in red blood cells (RBCs) has been extensively investigated because these cells are devoid of cellular organelles and thus are vulnerable to oxidative stress; RBCs contain no nucleoside diphosphate generating enzymes other than G6PD [5]. The pathogenesis of other diseases possibly involves G6PD deficiency; the toxicological mechanism of nitric oxide and benzopyrene also likely includes the interference of G6PD deficiency [6]. Cheng et al. investigated the growth-regulatory role of G6PD by using foreskin fibroblasts and found that G6PD deficiency predisposes human fibroblasts for retarded growth and reduces their replicative potential upon serial cultivation. G6PD is possibly involved in death signaling, in addition to its role in cellular proliferation and senescence. G6PD-deficient human fibroblasts undergo apoptosis after they are treated with an NO donor [7].

We previously performed serum peptidome analysis and found that G6PD is overexpressed in benzene-exposed mice exhibiting hematopoietic toxicities compared with that in normal control mice [8].

Benzene is a common organic solvent and chemical contaminant widespread in industrial and living environments [9]. Benzene was identified as a human carcinogen by the International Agency for Research on Cancer in 1982 [10]. Occupational chronic exposure can reduce peripheral white blood cells and can cause bone marrow depression and leukemia [11, 12]. The potential mechanisms of benzene toxicity are involved in oxidative damage, DNA mutation, and chromosome aberrations induced by benzene metabolites activated in liver and bone marrow [12, 13]. However, the mechanisms of benzene hematotoxicity and carcinogenicity in humans remain unknown.

In the liver, benzene is converted into its metabolites, namely, phenol, hydroquinone, catechol, and benzene triol [14, 15]. These metabolites are further oxidized into 1,4-benzoquinone (BQ) as catalyzed by myeloperoxidase in the bone marrow [16, 17]. Benzene metabolites can also be excreted when they conjugate with GSH or glucuronide as catalyzed by phase II metabolic enzymes, such as glutathione S-transferase pi-1 and uridine 5′-diphospho-glucuronosyltransferase 1A6 isozymes [18, 19].

G6PD is overexpressed in the plasma of mice exposed to benzene [8] and implicated in the maintenance of GSH in providing protection from oxidative damage. Considering these phenomena, we hypothesized that G6PD overexpression in benzene-exposed mice is a protective mechanism activated in response to oxidative stress induced by benzene exposure. Consequently, G6PD deficiency with insufficient GSH may induce a higher risk of benzene-induced toxicity than normal G6PD condition. To verify this hypothesis, we established a stable human leukemia K562 cell line through G6PD gene silencing and investigated whether G6PD inhibition can enhance BQ-induced oxidative damage in K562 cells.

## 2. Materials and Methods

### 2.1. Cell Culture

K562 cell line was purchased from Shanghai Cell Bank in Chinese Academy of Sciences (Shanghai, China). Cells were cultured in Iscove's Modified Dulbecco's Medium (IMDM, Gibco Ltd., Gaithersburg, USA) supplemented with 10% fetal bovine serum (FBS, Gibco Ltd., Gaithersburg, USA), 100 units/mL penicillin, and 100 units/mL streptomycin (Hyclone, Logan, UT) in a humidified 5% CO_2_ incubator at 37°C. In addition, 0.6 *μ*g/mL puromycin (Sigma Co., St. Louis, MO, USA) but not FBS, penicillin, and streptomycin was added into the IMDM cell culture medium of the G6PD inhibition and control cell line.

### 2.2. Transfection of siRNA Lentivirus of G6PD Gene in K562 Cell Line

**Figure 1:**
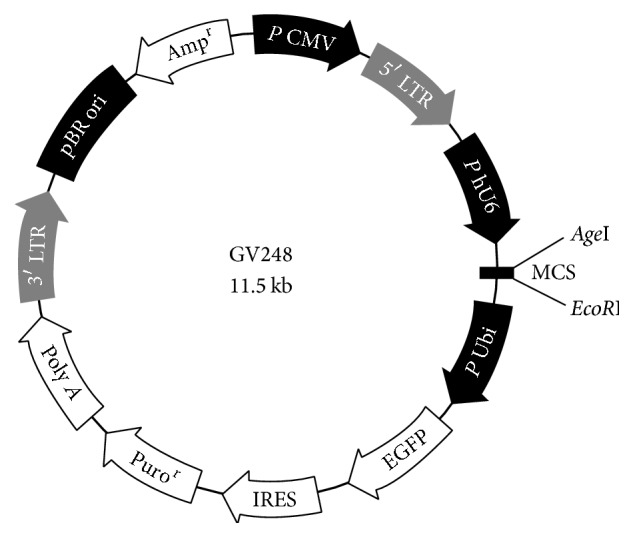
The siRNA vector map.

SiRNA lentivirus of G6PD gene, which included three target siRNA lentiviruses and a negative control siRNA lentivirus, was designed and synthesized by Shanghai Ji Kai Gene Technology Co., Ltd. Four lentiviruses were chemically modified with GFP fluorescence labeled and the vector information was as follows: hU6-MCS-Ubiquitin-EGFP-IRES-puromycin.  Figure 1 shows the vector map and Table 1 shows the siRNA sequence. The siRNA sequence of negative control is as follows: TTCTCCGAACGTGTCACGT.

### 2.3. Quantitative Real-Time PCR Analysis

Cells (2 × 10^5^/mL) were seeded into 6-well plates and treated with 0, 10, and 20 *μ*mol/L BQ for 24 h. Total RNA was extracted from the cells by using Trizol (Sigma Co., St. Louis, MO, USA). RNA content was measured by Nano Drop spectrophotometer and then reverse-transcribed using PrimeScript™ RT Master Mix (TaKaRa, Dalian, China) according to the manufacturer's protocol. Quantitative real-time PCR analyses of G6PD mRNA levels were performed in SYBR Green qPCR SuperMix (Toyobo, Osaka, Japan). The forward and reverse primers were as follows: G6PD, 5′-AAGAACGTGAAGCTCCCTGA-3′ and 5′-AATATAGGGGATGGGCTTGG-3′; *β*-actin, 5′-GCTCTGGCTCCTAGCACCAT-3′ and 5′-GCCACCGATCCACACAGAGT-3′. The amplification conditions were as follows: 95°C for 5 min, 40 cycles at 95°C for 15 s, and 60°C for 1 min. The melting curve conditions were as follows: 15 s at 95°C, 1 min at 60°C, and 15 s at 95°C.

### 2.4. Western Blot Analysis

Cells (2 × 10^5^/mL) were seeded into 6-well plates and treated with different BQ concentrations (0, 10, and 20 *μ*mol/L) for 24 h. The cells were washed twice with PBS and then lysed in RIPA lysis buffer (1 mM PMSF; Beyotime Biotechnology, Nantong, China) on ice for 10 min, sonicated for 5 s, and centrifuged at 12000 ×g for 10 min at 4°C. Protein concentration in the supernatant was assayed by Pierce BCA Protein Assay Kit (Thermo Fisher Scientific, Rockford, USA). The protein was mixed with 6*∗*SDS sample buffer and boiled for 5 min. Equal amounts of protein were electrophoresed on 10% SDS-PAGE and then electroblotted onto polyvinylidene fluoride membranes. The membrane was incubated overnight at 4°C with anti-G6PD polyclonal antibodies (RbpAb to G6PD; Abcam, Cambridge, UK) and anti-*β*-actin polyclonal antibodies (mouse monoclonal IgG; Santa Cruz, CA, USA). Secondary goat anti-rabbit IgG or goat anti-mouse IgG (Santa Cruz, CA, USA) was used according to the species of primary antibody. Chemiluminescence was measured by a chemiluminescent imaging system (Tanon-5200, Shanghai, China) using Immobilon™ Western Chemiluminescent HRP Substrate (Millipore Corporation, Billerica, USA).

### 2.5. G6PD Activity Assay

G6PD activity was detected using G6PD activity Assay Kit (Suzhou Comin Biotechnology Co., Ltd.) according to the manufacturer's protocol. Cells (3 × 10^5^/mL) were seeded into 6-well plates and treated with different BQ concentrations (0, 10, and 20 *μ*mol/L) for 24 h. The cells were washed with PBS. The samples were supersonic. The cells were centrifuged at 8000 ×g for 10 min at 4°C. The supernatant was used for activity determination. Absorbance was measured at 340 nm using a microplate reader.

### 2.6. NADPH/NADP Assays

The concentrations of NADP and NADPH were measured by ELISA kit (SenBeiJia Co., Ltd., Nanjing, China) according to the manufacturer's instructions. Briefly, cells were seeded into 6-well plates and treated with BQ at the final concentrations (0, 10, and 20 *μ*mol/L) for 6 h. The cells were washed with PBS and then frozen and thawed twice with liquid nitrogen in a water bath at 37°C. The cells were centrifuged at 3000 ×g for 20 min at 4°C. The supernatant was used for GSH and GSSG determination. Absorbance was measured at 450 nm using a microplate reader.

### 2.7. GSH and GSSG Assays

GSH and GSSG contents were detected using GSH and GSSG Assay Kit (Beyotime Biotechnology, Nantong, China) according to the manufacturer's protocol. Cells (2 × 10^5^/mL) were seeded into 6-well plates and treated with different BQ concentrations (0, 10, and 20 *μ*mol/L) for 24 h. The cells were washed with PBS, and protein removal agent was added. The samples were frozen and thawed twice with liquid nitrogen in a water bath at 37°C. The cells were centrifuged at 10000 ×g for 10 min at 4°C. The supernatant was used for NADPH and NADP determination. Absorbance was measured at 450 nm using a microplate reader.

### 2.8. ROS Detection

Intracellular ROS level was detected using dihydroethidium (DHE; Keygen Biotech, Nanjing, China). Briefly, the cells were treated with BQ (0, 10, and 20 *μ*mol/L) for 6 h and incubated with DHE in the dark for 20 min at 37°C. The cells were washed three times with IMDM and then analyzed by flow cytometry (BD Biosciences).

### 2.9. Comet Assay

OxiSelect™ Comet Assay Kit (3-Well Slides, 75 Tests; Cell Biolabs Inc., San Diego, USA) was used to evaluate oxidative DNA damage according to the manufacturer's protocol. Cells (2 × 10^5^/mL) were seeded into 6-well plates and treated with different BQ concentrations (0, 10, and 20 *μ*mol/L) for 24 h. Briefly, individual cells were mixed with molten agarose at 1 : 10 ratio (v/v) before placing onto OxiSelect comet slide (75 *μ*L/well). These embedded cells were subsequently treated with a prechilled lysis buffer and alkaline solution for 60 and 30 min at 4°C in the dark to relax and denature the DNA, respectively. Finally, the samples were electrophoresed in a horizontal chamber on alkaline electrophoresis solution to separate intact DNA from damaged fragments. Following electrophoresis, the samples were dried, stained with a DNA dye (100 *μ*L/well), and visualized through epifluorescence microscopy by using an FITC filter. The comet assay results were analyzed by casplab1.2.3 software.

### 2.10. Apoptosis Assay

The apoptotic rate was detected by Key Fluor 647-Annexin V/7AAD Kit (Keygen Biotech, Nanjing, China) through flow cytometry according to the manufacturer's instructions. Briefly, cells (2 × 10^5^/mL) were seeded into 6-well plates and treated with different BQ concentrations (0, 10, and 20 *μ*mol/L) for 24 h. The cells were washed with PBS and resuspended in binding buffer containing Annexin V-APC and 7-AAD. The cells were incubated at room temperature for 15 min in the dark and then analyzed by flow cytometry (BD Biosciences).

### 2.11. Cell Cycle Analysis

Flow cytometry was performed to determine cell cycle distribution using a cell cycle analysis kit (Suzhou Yue Ya Biological Technology Co., Ltd., Suzhou, China) according to the manufacturer's instructions. Cells (2 × 10^5^/mL) were seeded into 6-well plates and treated with 0, 10, and 20 *μ*mol/L BQ for 24 h and then analyzed through flow cytometry (BD Biosciences).

### 2.12. Statistical Analyses

SPSS 19.0 was used for data analysis. Data were expressed as mean ± SD. Comparisons were performed using one-way ANOVA followed by Dunnett's test; when variance was not homogeneous, multiple comparisons were performed using Dunnett's T3 test. Differences between two kinds of cells were determined by independent two-tailed *t*-test. Differences were considered significant at *P* < 0.05.

## 3. Results

### 3.1. Regulation of BQ-Induced G6PD Expression in K562 Cell Line

**Figure 2:**
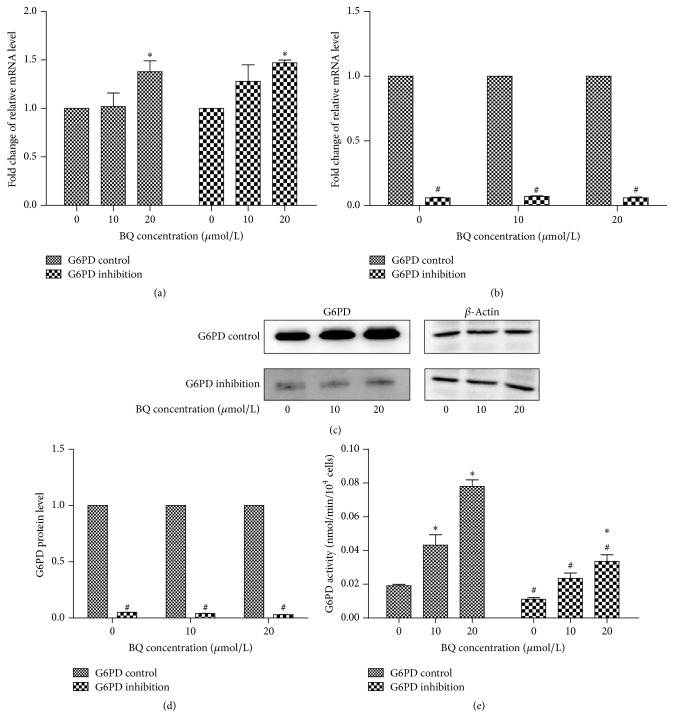
mRNA, protein expression levels, and enzyme activity of G6PD in G6PD inhibition cells and control cells exposed to BQ. (a) Fold change of relative mRNA level by 10 and 20 *μ*mol/L BQ treatment compared with 0 *μ*mol/L BQ in G6PD control and inhibition cells. (b) Fold change in relative mRNA in G6PD inhibition cells compared with that in control cells at each BQ concentration. (c) Protein level of G6PD as determined by Western blot analysis. (d) Fold change in protein level in G6PD inhibition cells compared with that in control cells at each BQ concentration. (e) G6PD activity in G6PD inhibition cells and control cells at each BQ concentration. ^*∗*^ *P* < 0.05 compared with 0 *μ*mol/L group; ^#^ *P* < 0.05 compared with G6PD control.

After several freeze-thaw cycles, G6PD inhibition and G6PD control cells were morphologically normal and their fluorescent protein was stably expressed. RT-PCR results showed that G6PD could be significantly upregulated in G6PD control and inhibition K562 cells exposed to BQ (*P* < 0.05; Figure 2(a)); in addition, relative G6PD mRNA expression in G6PD inhibition cells significantly decreased compared with that in G6PD control cells at each BQ concentration. The mRNA level also significantly increased in G6PD inhibition cells exposed to 20 *μ*mol/L BQ (*P* < 0.05; Figure 2(b)). The result of Western blot showed that the relative G6PD protein level in G6PD inhibition cells significantly decreased compared with that in control cells at each BQ concentration (*P* < 0.05; Figures 2(c) and 2(d)). The results of G6PD activity showed that the G6PD activity in G6PD inhibition cells (0.111 ± 0.001, 0.023 ± 0.003, and 0.034 ± 0.004) remarkably decreased compared with those in G6PD control cells (0.019 ± 0.001, 0.043 ± 0.006, and 0.078 ± 0.004) at each concentration of BQ (*P* < 0.05; Figure 2(e)).

### 3.2. Influence of G6PD Inhibition on NADPH/NADP and GSH/GSSG Ratio under BQ Exposure in K562 Cell Line

**Figure 3:**
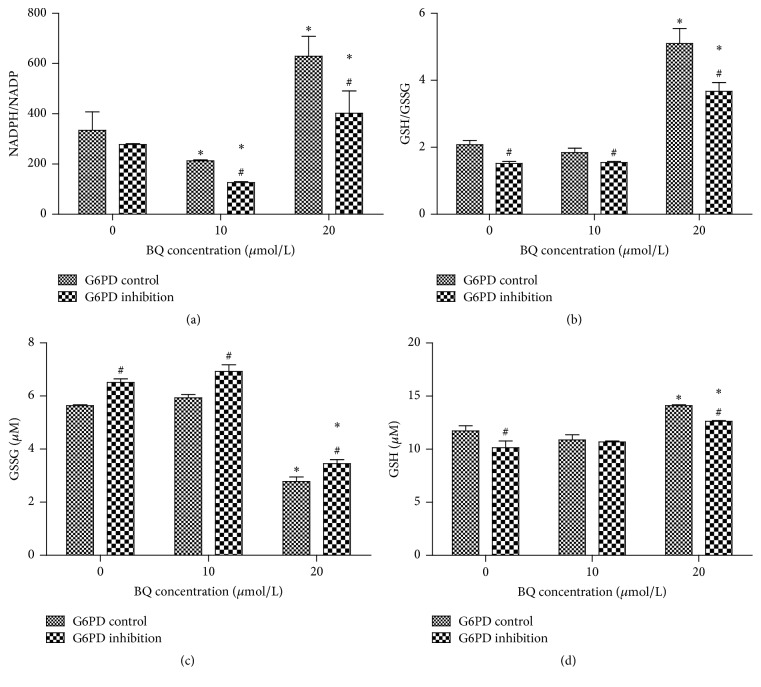
NADPH/NADP and GSH/GSSG ratio in G6PD control and G6PD inhibition. (a) Ratio of NADPH/NADP in G6PD inhibition cells and control cells exposed to BQ. (b) Ratio of GSH/GSSG in G6PD inhibition cells and control cells exposed to BQ. (c) GSSG in G6PD inhibition cells and control cells exposed to BQ. (d) GSH in G6PD inhibition cells and control cells exposed to BQ. ^*∗*^ *P* < 0.05 compared with 0 *μ*mol/L group; ^#^ *P* < 0.05 compared with G6PD control.

The results of colorimetric assay showed that the NADPH/NADP ratios in G6PD inhibition cells (277.73 ± 11.11, 126.28 ± 4.17, and 402.03 ± 89.14) remarkably decreased compared with those in G6PD control cells (334.44 ± 73.20, 212.73 ± 9.38, and 629.06 ± 79.37) at 10 and 20 *μ*mol/L BQ (*P* < 0.05; Figure 3(a)). The results also showed that the GSH/GSSG ratios in G6PD inhibition cells (1.52 ± 0.12, 1.55 ± 0.08, and 3.67 ± 0.26) remarkably decreased compared with those in G6PD control cells (2.08 ± 0.14, 1.84 ± 0.17, and 5.10 ± 0.45) at each BQ concentration (*P* < 0.05; Figure 3(b)).

### 3.3. Effect of G6PD Inhibition on BQ-Induced ROS Level in K562 Cell Line

**Figure 4:**
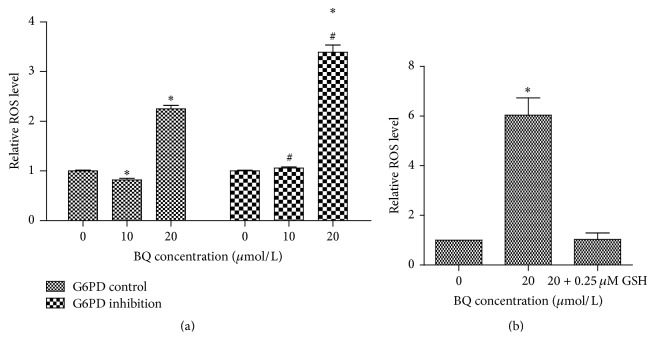
Relative ROS level in G6PD control and G6PD inhibition cells. (a) Relative ROS level in G6PD control and G6PD inhibition cells exposed to 10 and 20 *μ*mol/L BQ. (b) Relative ROS level in G6PD inhibition cells exposed to 20 *μ*mol/L BQ and treated with 0.25 *μ*mol/L GSH. ^*∗*^ *P* < 0.05 compared with 0 *μ*mol/L group; ^#^ *P* < 0.05 compared with G6PD control.

Fluorescence intensity was observed after treatment with different BQ concentrations for 6 h. The relative ROS level remarkably decreased first (0.82 ± 0.007) and then increased (2.25 ± 0.09) compared with that in 0 *μ*mol/L group in G6PD control cells (*P* < 0.05). The relative ROS level slightly increased first (1.06 ± 0.05) and then remarkably increased (3.39 ± 0.16) compared with that in 0 *μ*mol/L group in G6PD inhibition cells (*P* < 0.05). The relative ROS level was statistically increased at 10 and 20 *μ*mol/L group in G6PD inhibition cells compared with that in control cells (Figure 4(a)). Then the G6PD inhibition cells exposed to 20 *μ*mol/L BQ were treated with 0.25 *μ*mol/L GSH and the relative ROS level was significantly decreased from 6.04-fold to 1.03-fold (Figure 4(b)).

### 3.4. Effect of G6PD Inhibition on BQ-Induced DNA Damage in K562 Cell Line

**Figure 5:**
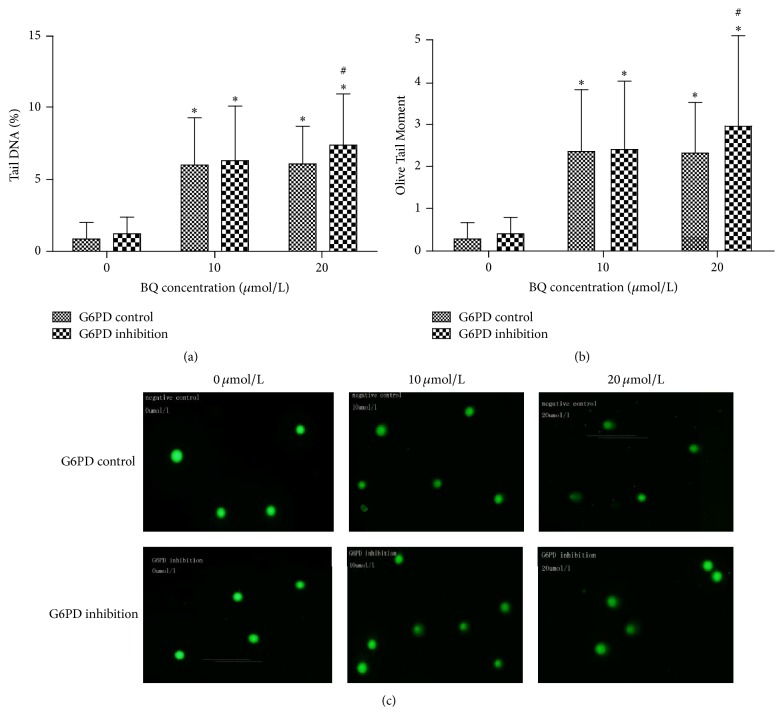
Effect of BQ on DNA damage in G6PD control and G6PD inhibition cells. The cells were treated with 0, 10, and 20 *μ*mol/L BQ for 24 h and then were analyzed under epifluorescence microscope. Data were expressed as mean ± SD with 50 cells in each group. Tail DNA% and Olive Tail Moment were analyzed through comet assay. (a) Tail DNA% = 100 × Tail DNA Intensity/Cell DNA Intensity. (b) Olive Tail Moment = Tail DNA%  × Tail Moment Length. (c) Representative photographs obtained through epifluorescence microscopy using an FITC filter. ^*∗*^ *P* < 0.05 compared with 0 *μ*mol/L group; ^#^ *P* < 0.05 compared with G6PD control.

Comet assay results showed that the G6PD inhibition cells displayed DNA damage compared with G6PD control cells (Figure 5). Tail DNA% and Olive Tail Moment significantly increased under exposure to 20 *μ*mol/L BQ in G6PD inhibition cells compared with those in control cells (*P* < 0.05). In addition, Tail DNA% and Olive Tail Moment at 10 and 20 *μ*mol/L BQ significantly increased compared with those in 0 *μ*mol/L group in both G6PD inhibition and G6PD control cells (*P* < 0.05).

### 3.5. BQ-Induced Apoptosis of G6PD Control and G6PD Inhibition Cells

**Figure 6:**
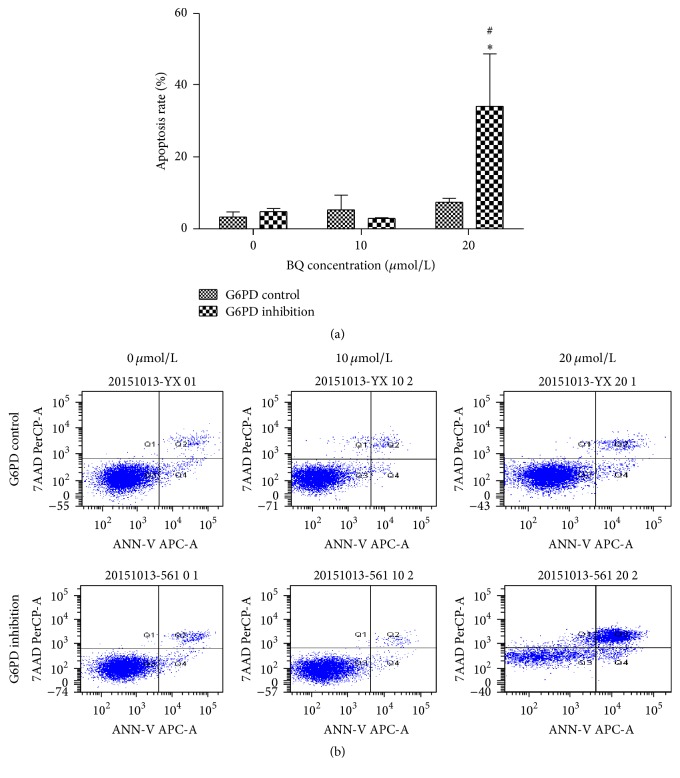
BQ-induced apoptosis in G6PD control and G6PD inhibition cells. (a) Apoptosis rate at 24 h induced by BQ. (b) Apoptosis illustrations by flow cytometry. ^*∗*^ *P* < 0.05 compared with 0 *μ*mol/L group; ^#^ *P* < 0.05 compared with G6PD control.

After treatment with different BQ concentrations for 24 h, flow cytometry was used to detect the apoptosis rate of G6PD control and G6PD inhibition cells. The results were shown in Figure 6. The corresponding apoptosis rates of G6PD control cells were 3.80% ± 1.65%, 5.23% ± 4.48%, and 7.27% ± 1.70%, whereas those of G6PD inhibition cells were 4.47% ± 1.68%, 2.83% ± 0.35%, and 34.23% ± 14.96%. The apoptosis rate increased with increasing BQ concentration compared with that of 0 *μ*mol/L group both in G6PD control and in G6PD inhibition cells. In addition, the apoptosis rate of the 20 *μ*mol/L group of G6PD inhibition cells significantly increased compared with that of G6PD control cells.

### 3.6. BQ-Induced Changes in Cell Cycle Progression in G6PD Control and G6PD Inhibition Cells

**Figure 7:**
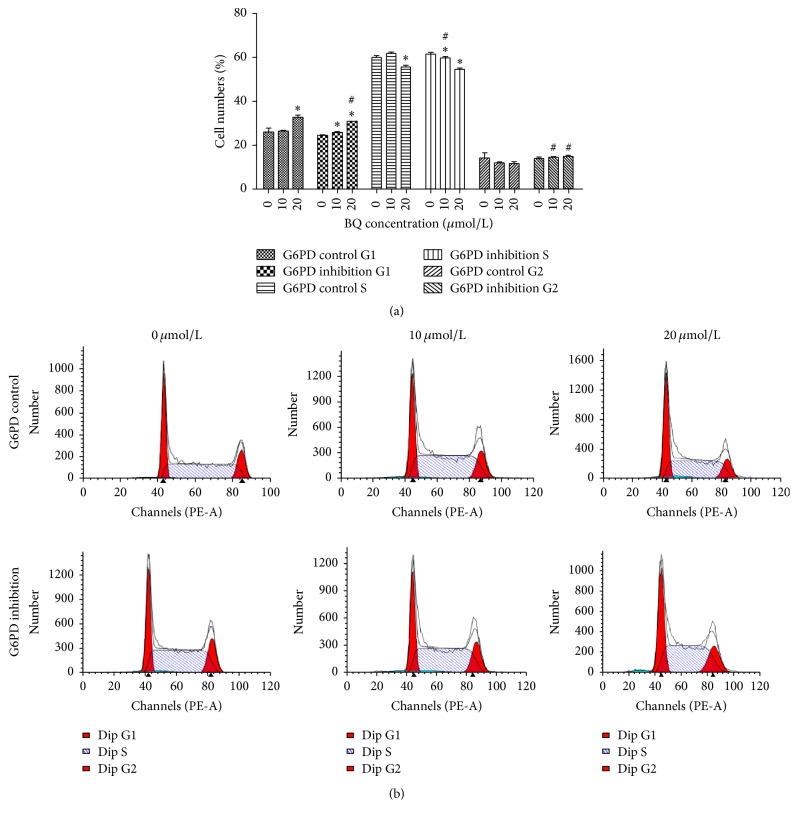
Changes in cell cycle progression in G6PD control and G6PD inhibition cells. (a) Distribution of G1, S, and G2 phases upon BQ exposure. (b) Typical chart by flow cytometry. ^*∗*^ *P* < 0.05 compared with 0 *μ*mol/L group; ^#^ *P* < 0.05 compared with G6PD control.

Increased number of cells in G1 phase and reduced numbers of cells in S phases were apparent in G6PD control cells and G6PD inhibition cells compared with those in 0 *μ*mol/L group. However, the trend for G2 phase varied between G6PD control cells and G6PD inhibition cells. In addition, the number of cells in G1 phase under exposure to 20 *μ*mol/L BQ significantly decreased in the G6PD inhibition cells compared with that in G6PD control cells. The number of cells in S phase under exposure to 10 *μ*mol/L BQ significantly decreased in G6PD inhibition cells compared with that in G6PD control cells. However, the number of cells in G2 phase among G6PD inhibition cells significantly increased compared with that among G6PD control cells when exposed to 10 and 20 *μ*mol/L BQ (*P* < 0.05; Figure 7).

## 4. Discussion

The significant upregulation of G6PD in G6PD inhibition and control cells treated with BQ confirmed that G6PD is involved in the mechanism of benzoquinone-induced toxicity. The mRNA level of G6PD at 20 *μ*mol/L BQ was 1.38-fold higher than that at 0 *μ*mol/L in control cells. In addition, G6PD was upregulated in G6PD inhibition cell line in a dose-dependent manner. The mRNA level, protein level, and activity of G6PD in G6PD inhibition cells at each BQ concentration were significantly lower than those in control cell line, suggesting the inadequate amount of G6PD in K562 cell line transfected with siRNA of G6PD.

Given that G6PD could produce NADPH to convert GSSG (oxidized glutathione) into GSH, GSH/GSSG ratios at each BQ concentration were significantly lower in G6PD inhibition cells than in control cell lines. This result demonstrated that GSH was reduced when G6PD expression was inhibited in K562 cell line. GSH detoxifies benzene, reacting with the oxidative metabolites of benzene to produce s-phenylmercapturic acid, which is the urinary biomarker of benzene exposure [20]. Tang et al. (2015) investigated RBCs and the results suggested that normal RBCs and G6PD-deficient RBCs differ in their responses to oxidants because G6PD-deficient cells cannot generate sufficient amount of NADPH to maintain GSH pool compared with normal cells [21]. Meanwhile, Ko et al. (2011) reported that the GSH/GSSG ratio in G6PD-deficient mice decreased by 34.2% (*P* = 0.005) compared with that in normal G6PD mouse, and methemoglobin level of a novel mouse model increased by 1.9-fold (*P* < 0.001) [22]. These results suggested that the inability of G6PD to produce sufficient amount of NADPH and to maintain GSH is associated with BQ-induced G6PD deficiency.

Metabolic oxidation of benzene could induce oxidative damage, which is involved in benzene-induced leukemia [23, 24]. Of all the metabolites of benzene, benzoquinone formation in bone marrow has been suggested as key step in oxidative damage [25]. This present study found that the relative ROS level at 10 and 20 *μ*mol/L BQ treatments was higher in G6PD inhibition group than in G6PD control groups. ROS, including superoxide anion radical, hydrogen peroxide, and hydroxyl radical, is mainly produced in normal cellular metabolism as an endogenous molecule [26]. However, excessive ROS production induced by xenobiotic exposure could induce oxidative stress, which could increase DNA damage and thus initiate carcinogenesis [27]. In this study, the results of comet assay showed that severe DNA damage occurred in G6PD inhibition cells compared with that in control cells at 20 *μ*mol/L BQ treatment, suggesting that BQ-induced inhibition of G6PD could increase DNA damage in K562 cell line. In addition to inducing ROS generation, BQ could form DNA-adducts that also cause DNA damage [28]. The present study found that DNA damage in control cells increased at 20 *μ*mol/L, in accordance with ROS level increasing at 20 *μ*mol/L BQ.

Exposure of mammalian cells to benzene could generate DNA mutations, such as insertions and deletions, as well as strand breaks, sister chromatid exchange, and apoptosis [29, 30]. DNA damage was enhanced in G6PD inhibition cell and subsequent increased apoptosis rate and caused cell cycle disorder. The apoptosis rate in G6PD inhibition cells significantly increased by 4.7-fold compared with that in G6PD control cells at 20 *μ*mol/L BQ. Fenga et al. found that chronic exposure to low-dose benzene can modulate signal transduction pathways activated by oxidative stress and is involved in cell apoptosis [31]. Epidemiologic, clinical, and laboratory data demonstrated that occupational chronic exposure could reduce peripheral aplastic anemia resulting from bone marrow depression [32–34]. In addition, G2 phase arrest can increase apoptosis and participate in DNA repair [35, 36]. In this work, the number of cells in G2 phase in G6PD inhibition group significantly increased compared with that in G6PD control group when exposed to BQ. The enhanced apoptosis rate and G2 cycle arrest in G6PD inhibition cell demonstrated that G6PD inhibition could enhance oxidative damage in K562 cell line.

## 5. Conclusion 

Benzene is a toxic contaminant widespread in industrial and living environments. G6PD can promote GSH regeneration to protect cells against oxidative damage and injury. In this study, G6PD was involved in the mechanism of BQ-induced toxicity. mRNA and protein levels increased in G6PD control and G6PD inhibition cells exposed to BQ. These findings confirmed that G6PD inhibition may not produce sufficient amounts of NADPH to maintain GSH during exposure to BQ. Thus, the relative ROS level was increased in G6PD inhibition cell line exposed to BQ. DNA damage in G6PD inhibition cells was severe. The apoptosis rate and G2 arrest rate were also increased in G6PD inhibition cell line. Thus, G6PD inhibition could enhance BQ-induced oxidative damage in K562 cells.

Considering the hematopoietic toxicity and carcinogenicity of benzene and G6PD deficiency affecting over 400 million individuals worldwide, we provided novel insights into the protective mechanisms of G6PD-deficient population against benzene poisoning. Further studies should be conducted to investigate whether G6PD deficiency can alleviate oxidative damage and hematopoietic toxicity in a G6PD mouse model and whether G6PD-deficient individuals exposed to benzene have high incidence of bone marrow depression and leukemia.

## Figures and Tables

**Table 1:** siRNA sequence of Homo G6PD gene.

Number	Accession	Target seq	CDS	GC (%)
G6PD-RNAi(21559-1)	NM_000402	TGATGAAGAGAGTGGGTTT	149⋯1786	42.11
G6PD-RNAi(21561-1)	NM_000402	ACAGATACAAGAACGTGAA	149⋯1786	36.84
G6PD-RNAi(21563-1)	NM_000402	AGTCGGATACACACATATT	149⋯1786	36.84
